# A cuproptosis-related LncRNA signature: Integrated analysis associated with biochemical recurrence and immune landscape in prostate cancer

**DOI:** 10.3389/fgene.2023.1096783

**Published:** 2023-02-24

**Authors:** Lei Ren, Xu Yang, Weifeng Wang, Hansen Lin, Guankai Huang, Zixiong Liu, Jincheng Pan, Xiaopeng Mao

**Affiliations:** ^1^ Department of Urology, The First Affiliated Hospital of Sun Yat-sen University, Sun Yat-sen University, Guangzhou, China; ^2^ Department of Urology, The Seventh Affiliated Hospital of Sun Yat-sen University, Sun Yat-sen University, Shenzhen, China

**Keywords:** cuproptosis, prostate cancer, lncRNA, biochemical recurrence, prognostic model, immune cell infiltration

## Abstract

**Background:** As a new form of regulated cell death, cuproptosis differs profoundly from apoptosis, ferroptosis, pyroptosis, and necroptosis. The correlation between cuproptosis and long non-coding RNAs (lncRNAs) has been increasingly studied recently. In this study, a novel cuproptosis-related lncRNA prognostic signature was developed to investigate biochemical recurrence (BCR) and tumor immune landscape in prostate cancer (PCa).

**Methods and Materials:** The transcriptome data and clinicopathologic information of PCa patients were downloaded from The Cancer Genome Atlas (TCGA). Pearson’s correlation analysis was applied to identify lncRNAs associated with cuproptosis. Based on Cox regression analysis and the least absolute shrinkage and selection operator (LASSO) regression analysis, we developed a cuproptosis-related lncRNA prognostic model (risk score) to predict the BCR of PCa patients. Additionally, we also constructed a nomogram with the risk score and clinicopathologic features. The biological function, tumor mutation burden (TMB), immune cell infiltration, expression levels of immune checkpoint genes, and anti-cancer drug sensitivity were investigated.

**Results:** We constructed and validated the cuproptosis-related lncRNA signature prognostic model (risk score) by six crlncRNAs. All patients were divided into the low- and high-risk groups based on the median risk score. The Kaplan–Meier (KM) survival analysis revealed that the high-risk group had shorter BCR-free survival (BCRFS). The risk score has been proven to be an independent prognostic factor of BCR in PCa patients. In addition, a nomogram of risk scores and clinicopathologic features was established and demonstrated an excellent predictive capability of BCR. The ROC curves further validated that this nomogram had higher accuracy of predicting the BCR compared to other clinicopathologic features. We also found that the high-risk group had higher TMB levels and more infiltrated immune cells. Furthermore, patients with high TMB in the high-risk group were inclined to have the shortest BCRFS. Finally, patients in the high-risk group were more susceptible to docetaxel, gefitinib, methotrexate, paclitaxel, and vinblastine.

**Conclusion:** The novel crlncRNA signature prognostic model shows a greatly prognostic prediction value of BCR for PCa patients, extends our thought on the association of cuproptosis and PCa, and provides novel insights into individual-based treatment strategies for PCa.

## Introduction

Prostate cancer (PCa), which accounts for over 1,400,000 new cases and 375,000 deaths globally in 2020, is the second most frequent kind of cancer and the fifth greatest cause of cancer-related mortality in men (Sung et al., 2021). For patients with localized PCa, the preferred treatment strategies are still radical prostatectomy (RP) and radiotherapy (RT) (Achard et al., 2021). Although, with adequate preferred treatments, more than 35% of patients will ultimately develop into biochemical recurrence (BCR) or clinical relapse (Freedland et al., 2007). Once patients have a BCR, poor prognosis and underlying clinical metastases are inevitable without timely detection and treatment, significantly reducing the overall survival and the quality of life (Lalonde et al., 2014; Xu et al., 2020). Therefore, early prediction is increasingly important to identify patients with a high risk of BCR. Although there are remaining specific biomarkers and clinical characteristics to assess the risk of BCR, including PSA level of pretherapy, tumor stage, and Gleason score (GS), these factors seem incapable of comprehensively evaluating the risk of BCR for patients (Kretschmer and Tilki, 2017; Mottet et al., 2021). Consequently, it is critical to identify an accurate risk model to predict BCR early and improve the prognosis of patients.

Long non-coding RNA (lncRNA) pertains to non-coding RNA with longer than 200 nucleotides and lacks a function to encode proteins. There are growing evidences that lncRNAs play a crucial role in tumor growth and metastasis through proliferation, migration, and invasion (Quinn and Chang, 2016; Liang et al., 2018; Chi et al., 2019; Statello et al., 2021), indicating that some specific lncRNAs can be potential biomarkers and targets for the treatment of many cancers. Meanwhile, lncRNAs also exert substantial impact on immune escape and the tumor microenvironment (TME) regulation, assisting tumor cells in evading immune surveillance (Quinn and Chang, 2016; Hu et al., 2019; Pi et al., 2021).

During the average growth and development of mammals, regulated cell death (RCD), as well-known as programmed cell death (PCD), plays a vital role in the evolution of organisms, stabilization of the internal environment, and development of multiple systems by a variety of biomacromolecules and signal amplification complexes (Galluzzi et al., 2018; Peng et al., 2022). Based on the occurrence mechanism, RCDs incorporate apoptosis, ferroptosis, necroptosis, and pyroptosis, which collaborate to maintain the stability of the cell cycle. However, abnormal RCDs have been proven to take part in various disease processes, including immune system dysfunction, developmental disorders of the body, neurodegeneration, and especially tumorigenesis (Tang et al., 2019). For patients with tumors, targeting RCD will provide an emerging therapeutic strategy (Liu W. et al., 2022; Peng et al., 2022). Therefore, developing a new model of RCDs would not merely improve our understanding of tumorigenesis but provide novel therapeutic targets for tumors. A recent study has revealed that cuproptosis is a novel cell death mechanism that differs from the abovementioned RCDs (Tsvetkov et al., 2022). Copper is a significant catalytic cofactor of several essential enzymes and plays a vital role in many metabolic processes (Scheiber et al., 2013). According to previous studies, copper at unbalanced concentrations could lead to the development and progression of many tumors, as well as cause the death of cells (Tisato et al., 2010; Shanbhag et al., 2021). Strikingly, Tsvetkov et al. found that the amassing of intracellular copper could induce aggregation of the lipoylated proteins and loss of iron-sulfur cluster protein in the TCA cycle, finally resulting in proteotoxic stress-regulated cell death, which was defined as cuproptosis (Tsvetkov et al., 2022). Previous studies have revealed that cuproptosis is closely related to the development and immune response of many cancers, such as clear cell renal cell carcinoma, bladder cancer, and hepatocellular carcinoma (Xu S. et al., 2022; Song et al., 2022; Zhang et al., 2022). However, very little is currently known about the correlation between cuproptosis-related lncRNAs and PCa in predicting the prognosis.

In this study, we analyzed the transcriptome data (RNA-seq) of PCa from the TCGA database to construct a prognostic model based on the cuproptosis-related lncRNA signature to predict biochemical recurrence (BCR). A nomogram was developed to improve the prognostic prediction value for PCa patients. Moreover, based on this prognostic model, we performed functional enrichment analysis, immune cell infiltration, and TMB to investigate underlying molecular mechanisms.

## Methods and materials

### Public data collection

We downloaded the RNA sequencing (RNA-seq) data and clinicopathologic data of PCa samples from The Cancer Genome Atlas (TCGA) database (https://portal.gdc.cancer.gov/repository). We utilized the Fragment Pre Kilobase Method (FPKM) platform to gather the RNA-seq data. The log transformation was applied to normalize the RNA-seq. To avoid statistical bias as much as possible, we removed samples without complete clinical information. In total, we included 455 patients in our study with 404 tumor samples and 51 normal samples. In a 1:1 ratio, all PCa patients randomly split into the training set and the test set.

### Identification of differentially expressed cuproptosis-related lncRNAs

We obtained ten cuproptosis-related genes (including FDX1, LIPT1, DLD, LIAS, DLAT, PDHA1, PDHB, MTF1, GLS, and CDKN2A) from previous studies (Tsvetkov et al., 2022), and explored their expression levels between tumor samples and normal samples with cut-off criteria of |log_2_FC|≥1 and p. adj. <0.05 using the “DESeq2” R package and prognostic values in PCa patients by the univariate Cox regression analysis (*p* < 0.05). Pearson’s correlation analysis was applied to identify cuproptosis-related lncRNAs (crlncRNAs) with the filter criteria |Pearson R| > 0.3 and *p* < 0.001. Then, we screened differentially expressed lncRNAs between tumor samples and normal samples with cut-off criteria of |log_2_FC|≥1 and p. adj. <0.05 using the “DESeq2” R package *via* the Wilcoxon test (Liu et al., 2021). Ultimately, the overlapped lncRNAs were recognized as differentially expressed crlncRNAs (DE-crlncRNAs), and the forest plot was drawn.

### Establishment and evaluation of the cuproptosis-related lncRNA signature prognostic model

The DE-crlncRNAs expression and BCR free survival were matched. The univariate Cox regression analysis was utilized to identify prognosis-related lncRNAs among DE-crlncRNAs (*p* < 0.05). We performed the LASSO Cox regression analysis using the “survival” and “glmnet” R package in the training set to further screen optimal prognosis-related DE-crlncRNAs. Then, we developed a crlncRNA signature prognostic risk model based on optimal lncRNAs by multivariate Cox regression. Finally, the risk score for each patient was calculated based on the formula below:

$$Risk\;score=\overset{n}{\underset{i=1}{\sum }}\left(coefi\left(lncRNAi\right)\;*\;\exp\left(lncRNAi\right)\right)$$

The coefi refers to the coefficient, and exp refers to the normalized expression level of lncRNAs.

We used the median score as the cutoff point to divide all PCa patients into the low-risk and high-risk groups. The Kaplan–Meier (KM) survival analysis was performed to compare the BCRFS between the low- and high-risk groups in the training, test and whole sets using the “survival” R package. Then, we evaluated the predictive accuracy of the prognostic risk model by ROC curves analysis using the “ROC” and “rms” R packages (McHugh, 2013). The area under the curve (AUC) was utilized to quantify the ROC curve.

The chi-square test was applied to assess the correlations between the prognostic risk model and clinicopathologic features. We also performed univariate and multivariate Cox regression analysis to explore whether the risk score was an independent prognostic risk factor for BCR among clinicopathologic features.

### Construction and evaluation of the nomogram

With the “rms” and “survival” packages in R, a nomogram of the risk score and clinicopathologic features was constructed to predict the BCR in PCa patients. To evaluate the nomogram’s effectiveness, the Calibration curve and time-dependent ROC curves analysis was performed.

### Functional enrichment analysis

We conducted gene set enrichment analysis (GSEA) to analyze potentially enriched pathways between the two groups using GSEA software (version 4.3.2, http://www.gsea-msigdb.org/gsea/index.jsp). *P*. adj. < 0.05 and simulated value = 1,000 were considered statistically significant.

### Analysis of tumor mutation burden and immune cell infiltration

We obtained the somatic mutation data of PCa patients from the TCGA database. Then, we analyzed and assessed TMB using the “maftools” R package to explore difference of TMB between the low- and high-risk groups. Based on the median value of TMB, patients were divided into the low and high TMB groups, and the K-M survival analysis was performed. Besides, we calculated correlations between the risk score and TMB by Pearson correlation analysis. We utilized CIBERSORT algorithm to evaluated the immune cells levels of the two groups (Chen et al., 2018). Furthermore, we applied ssGSEA algorithm to quantify the subgroups of the infiltrating immune cells between the two groups.

### Drug sensitivity prediction of the risk model

Based on the “pRRophetic” R package, we predicted the IC50 values of common chemotherapeutic drugs for the low- and high-risk groups on the Genomics of Drug Sensitivity in Cancer (GDSC; https://www.cancerrxgene.org/) database. We collected the immune checkpoint genes from previous studies (Kgatle et al., 2021) and compared expressed levels of immune checkpoint genes by Wilcoxon test analysis (*p* < 0.05) between the two groups to the clinical significance of the risk model in immunotherapy.

### Cell culture

Human prostate cancer cells PC3, DU145, and LNCAP and human normal prostate epithelium cell line RWPE-1 were purchased from Procell (Procell Life Science and Technology Co., Ltd.). Cells were cultured in RPMI-1640 medium (Invitrogen) mixed with 10% FBS. The incubator was set as a water-saturated atmosphere with 5% CO_2_ at 37°C.

#### Quantitative real-time PCR (qRT-PCR)

The TRIzol (Invitrogen, United States) reagent was utilize to extract total cellular RNA based on the protocol. RNA was reverse transcribe to cDNA by the PrimeScript RT reagent kit (EZBioscience, China). EZBioscience 2 × SYBR Green qPCR Master Mix (EZBioscience, China) conducted the procedure. Primers for qRT-PCR were provided by TSINGKE (Beijing TSINGKE Biotech Co., Ltd., China) and shown in Supplementary Table S1. ACTB was chosen for the internal reference. Expression levels of lncRNAs were measured as 2^−ΔΔCT^.

### Statistical analysis

R software (ver. 4.0.1) was utilized to conduct data analysis and visualize the results. Pearson correlation analysis was applied to calculated the correlation coefficient between variables. The log-rank test was performed to determine statistically significant differences between K-M curves. The heatmap in our study were constructed using the “heatmap” R package. SPSS version 25 (IBM SPSS Statistics for Windows, version 25.0, Inc., Chicago, IL, United States) was used to perform the univariate and multivariate Cox regression analysis to confirm the independent prognostic risk factor of the risk score. GraphPad Prism 9.0 (GraphPad, La Jolla, CA, United States) was utilized to draw forest plots. Statistical significance was defined as *p*-value <0.05 when there is no special description for above methods.

## Results

### Differentially expressed cuproptosis-related genes in PCa

**FIGURE 1:**
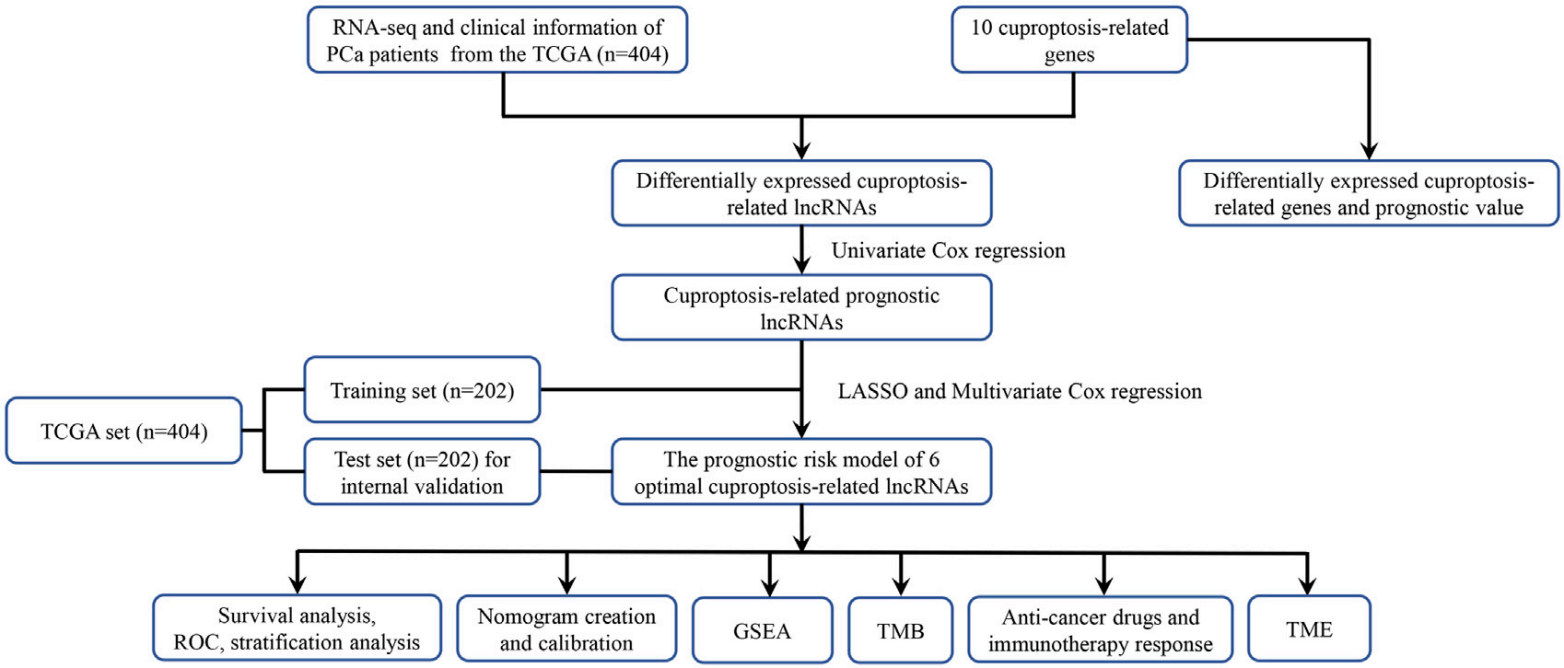
The flowchart of this study.

**FIGURE 2:**
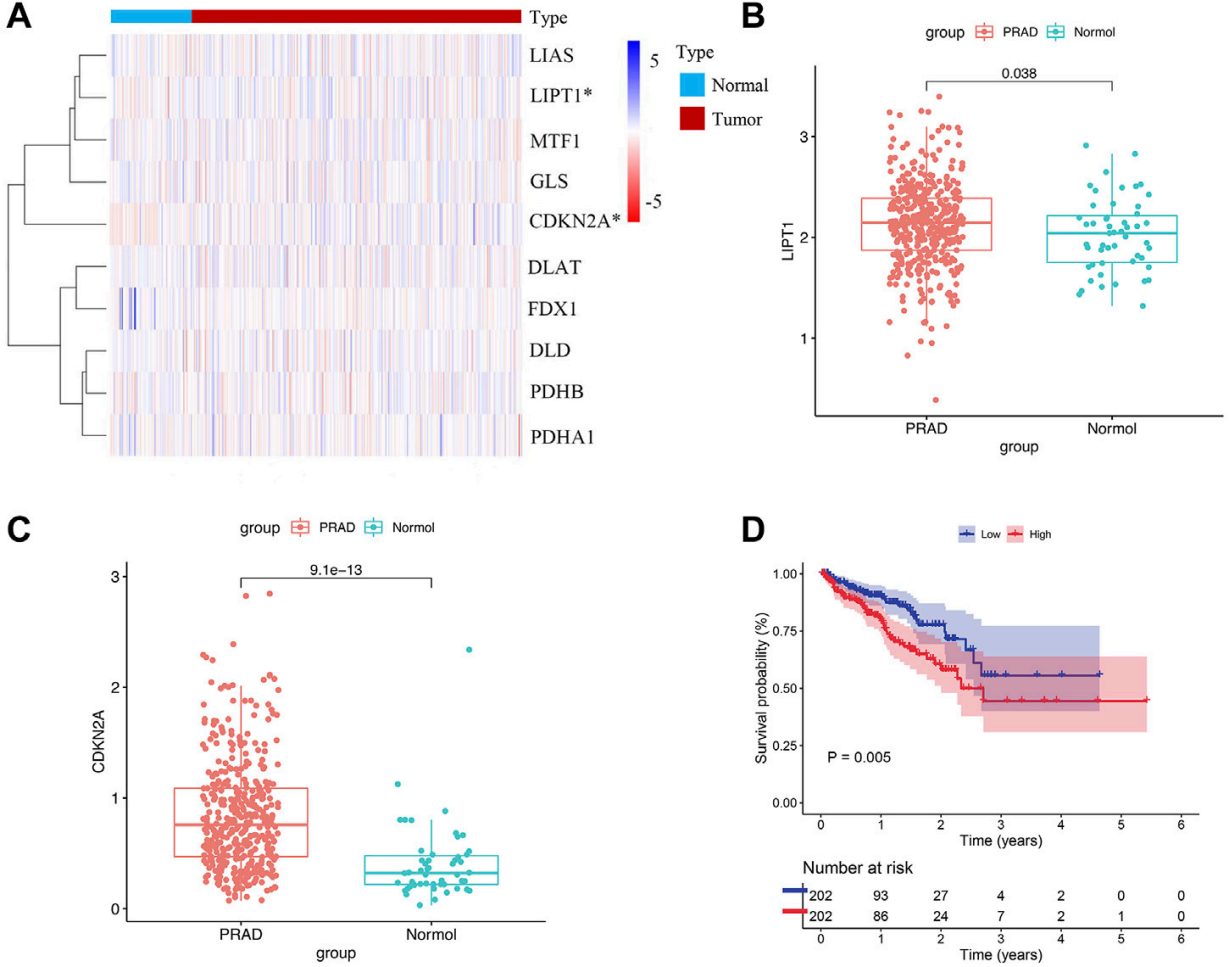
Cuproptosis-related genes expressed in PCa patients and prognostic value. **(A)** Expression levels of ten genes. **(B, C)** The comparison of LIPT1 and CDKN2A between PCa tissues and normal tissues. **(D)** The prognostic value of CDKN2A.

The flowchart of our study was displayed in Figure 1. Two of ten cuproptosis-related genes were differentially expressed between 404 PCa samples and 51 normal tissues, including LIPT1 and CDKN2A (Figures 2A,B). It was found that patients with high CDKN2A expression had worse clinical outcomes (shorter BCRFS), as shown in Figure 2C. These results indicated that cuproptosis might involve in the prognostic development of PCa patients.

### Identification of the differentially expressed crlncRNAs

**FIGURE 3:**
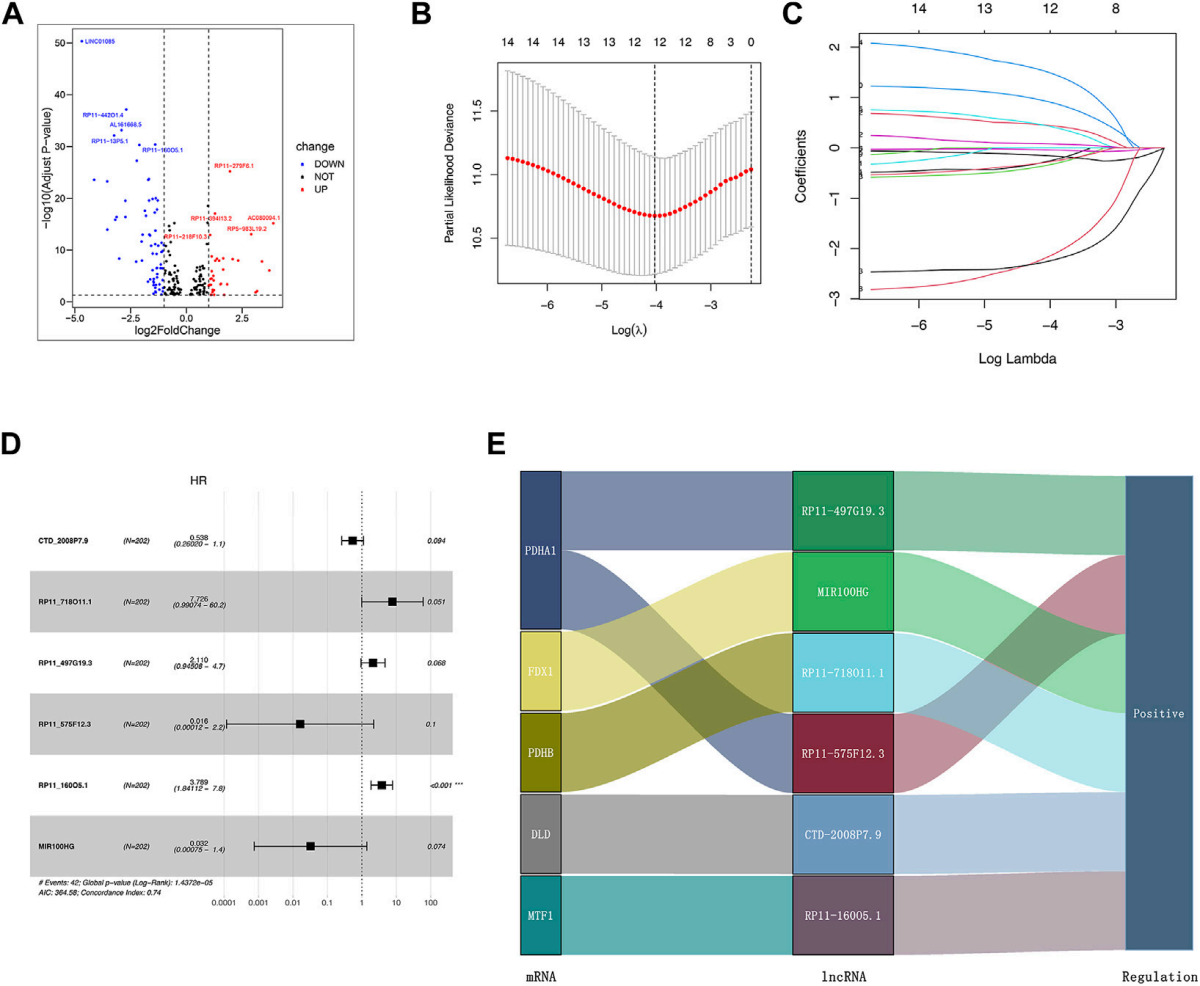
Establishment of the crlncRNA signature prognostic model. **(A)** The volcano plot of 101 DE-crlncRNAs. **(B)** The 10-fold cross-validation for variable selection in the LASSO algorithm. **(C)** The LASSO coefficients of six crlncRNAs. **(D)** Multivariate Cox regression showed six crlncRNAs. **(E)** The Sankey diagram indicated the correlations of the cuproptosis-related genes and six crlncRNAs.

We identified 1,187 differentially expressed lncRNAs from the TCGA database with the cut-off criteria of |log_2_FC|≥1 and the false discovery rate (FDR) < 0.05. Among 1,187 lncRNAs, 101 lncRNAs were associated with the cuproptosis by Pearson’s correlation analysis and codetermined as DE-crlncRNAs, among which 66 lncRNAs were downregulated and 35 lncRNAs were upregulated (Supplementary Table S2, Figure 3A). Through the univariate Cox regression analysis, we ultimately selected 14 prognosis-related DE-crlncRNAs (Supplementary Figure S1).

### Establishment of the crlncRNA signature prognostic model

Firstly, we randomly divided all PCa patients from the TCGA database into the training and test sets. The clinicopathologic features of these patients were presented in Table 1. In order to prevent overfitting, we performed LASSO Cox regression analysis in the training set to further screen six optimal prognosis-related DE-crlncRNAs and calculated their coefficients (Figures 3B,C). Then, the prognostic risk model of six lncRNAs was established by multivariate Cox regression (Figure 3D). The Sankey diagram revealed the correlation of the cuproptosis-related genes and six lncRNAs (Figure 3E). Interestingly, we found that RP11_160O5.1 was an independent prognostic risk factor for BCR in PCa patients. The Sankey diagram revealed the correlation of the cuproptosis-related genes and six lncRNAs. Finally, the risk score for each patient was calculated with the formula below:

$$Risk\;Score=RP11-497G19.3\times \left(0.7466\right)+RP11-575F12.3\times \left(-4.1249\right)+RP11-718O11.1\times \left(2.0446\right)+MIR100HG\times \left(-3.4310\right)+CTD-2008P7.9\times \left(-0.6198\right)+RP11-160O5.1\times \left(1.3322\right)$$

**TABLE 1:** Clinicopathologic features of 404 PCa patients from the TCGA database.

Variable	Training set	Test set	Whole set
	(*n* = 202)	(*n* = 202)	(*n* = 404)
**Age (years)**			
<65	144	142	286
≥65	58	60	118
**T stage**			
T2	78	62	140
T3	120	134	254
T4	4	6	10
**N stage**			
N0	165	165	330
N1	37	37	74
**Metastasis**			
M0	190	186	376
M1	12	16	28
**Gleason score**			
6	13	10	23
7	100	101	201
8	31	26	57
9	55	65	120
10	3	0	3

### Evaluation and validation of the crlncRNA prognostic risk model

**FIGURE 4:**
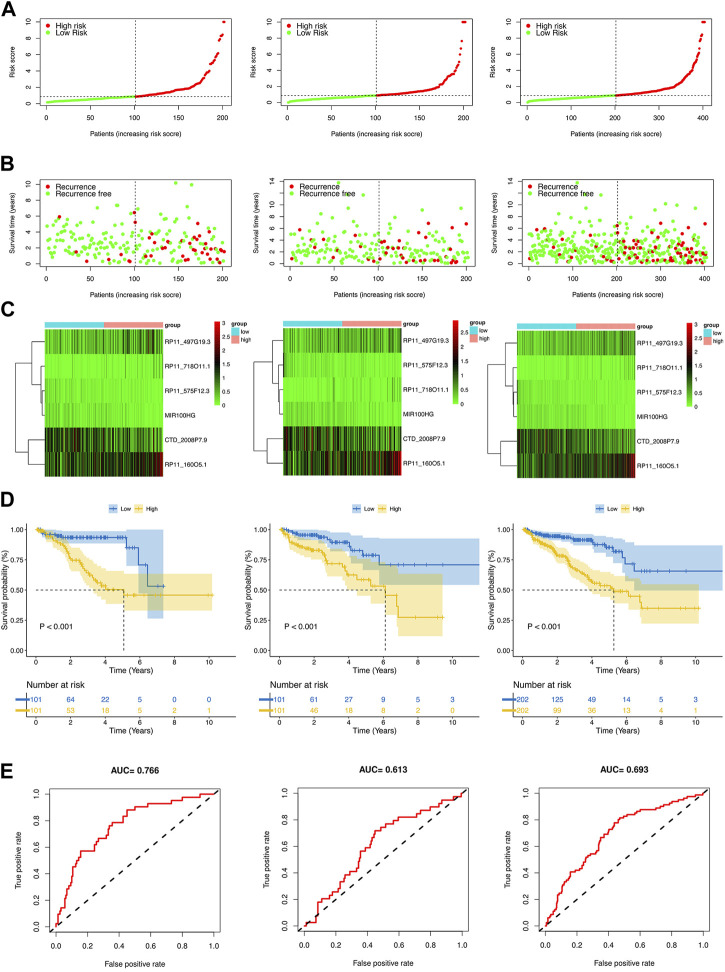
Prognosis value of the risk model in different sets. **(A)** The risk score distribution of PCa patients based on crlncRNAs in the training, test, and whole sets, respectively. **(B)** BCRFS and BCR status between the low- and high-risk groups in the training, test, and whole sets, respectively. **(C)** The heatmap of six crlncRNAs expression in the training, test, and whole sets, respectively. **(D)** Kaplan-Meier survival analysis of BCRFS of patients in the training, test, and whole sets, respectively. **(E)** ROC curve analysis of the risk model in the training, test, and whole sets, respectively.

By using the median risk score as the cut-off point in the training, test, and whole sets, all patients were divided into the low-risk and high-risk groups (Figures 4A,B). The survival curves revealed that patients in the high-risk group tended to have significantly shorter BCRFS than in the low-risk group, which was validated by the test set and whole set (Figure 4C). The heatmaps of the expression profiles of six prognosis-related DE-crlncRNAs in three sets were shown in Figure 4D. The AUCs for the training set, test set, and whole set of time-dependent ROC curve analysis were 0.766, 0.613, and 0.693, respectively (Figure 4E). These results suggested that the prognostic risk model exhibited good sensitivity and better power of this model to predict BCR for PCa patients.

### The clinical significance of the prognostic model

**FIGURE 5:**
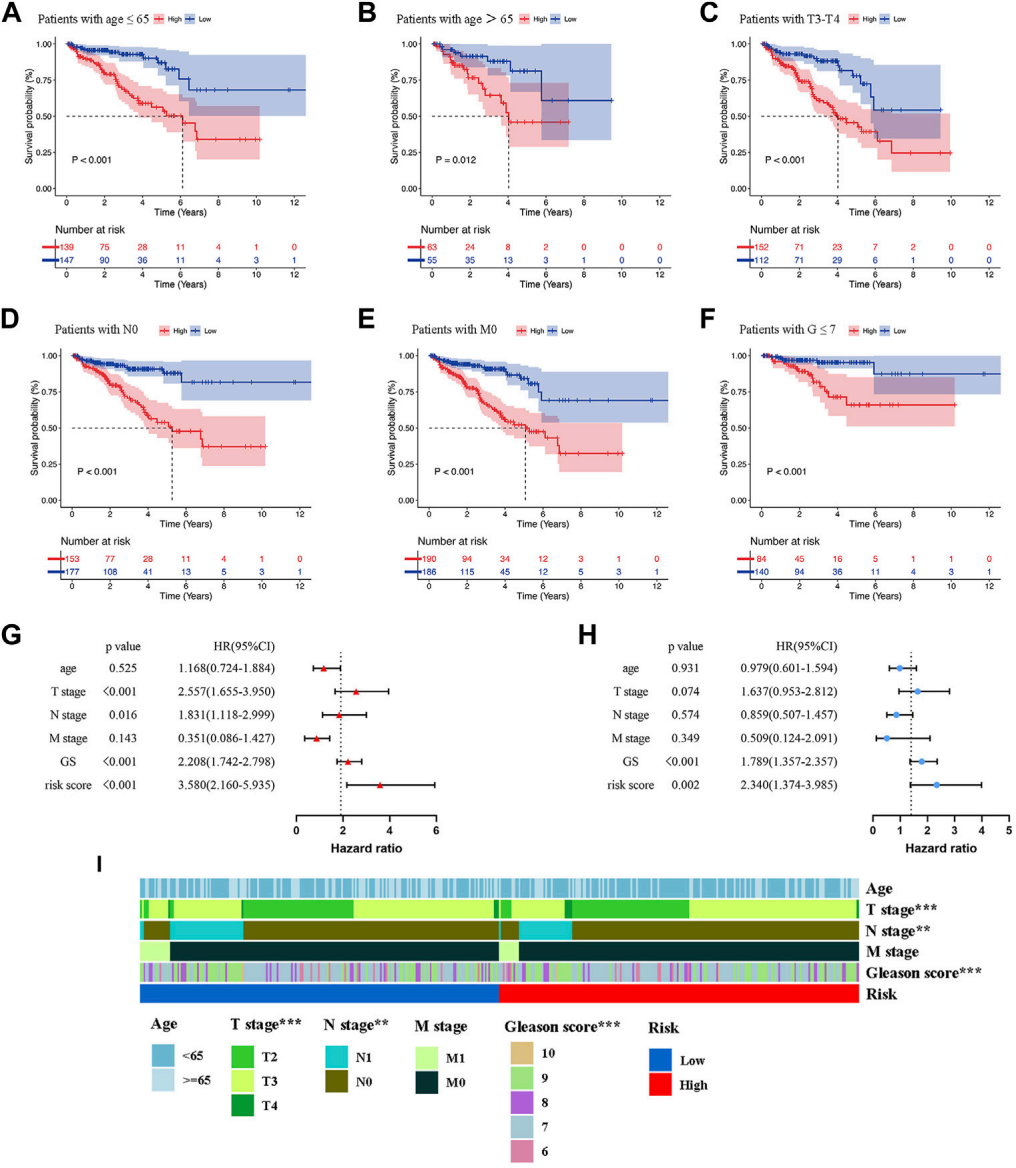
Kaplan-Meier survival analysis of BCR in the low- and high-risk groups for patients with age≤65 **(A)**, patients with age>65 **(B)**, patients with T3-T4 **(C)**, patients with N0 **(D)**, patients with M0 **(E)**, patients with G ≤ 7 **(F)**. Univariate **(G)** and Multivariate Cox regression **(H)** confirmed that the risk score and GS were independent prognostic factors of BCR of PCa patients. **(I)** The correlations between the prognostic model and clinicopathologic variables (age, TNM stage, and Gleason score). **p < 0.01; ***p < 0.001.

To evaluate the clinical significance of the prognostic model, patients of the whole set were divided into different subgroups by variable clinicopathological features (age, T stage, N stage, M stage, and Gleason score) *via* stratification analysis (Figures 5A–F). The K-M survival curves revealed that patients in the high-risk group had shorter BCRFS for different classifications, except for GS > 7, T2 stage, and N1 stage (Supplementary Figure S2). The results suggested that our model had broad applicability to clinically predict the BCR for PCa patients with different clinicopathological features.

**FIGURE 6:**
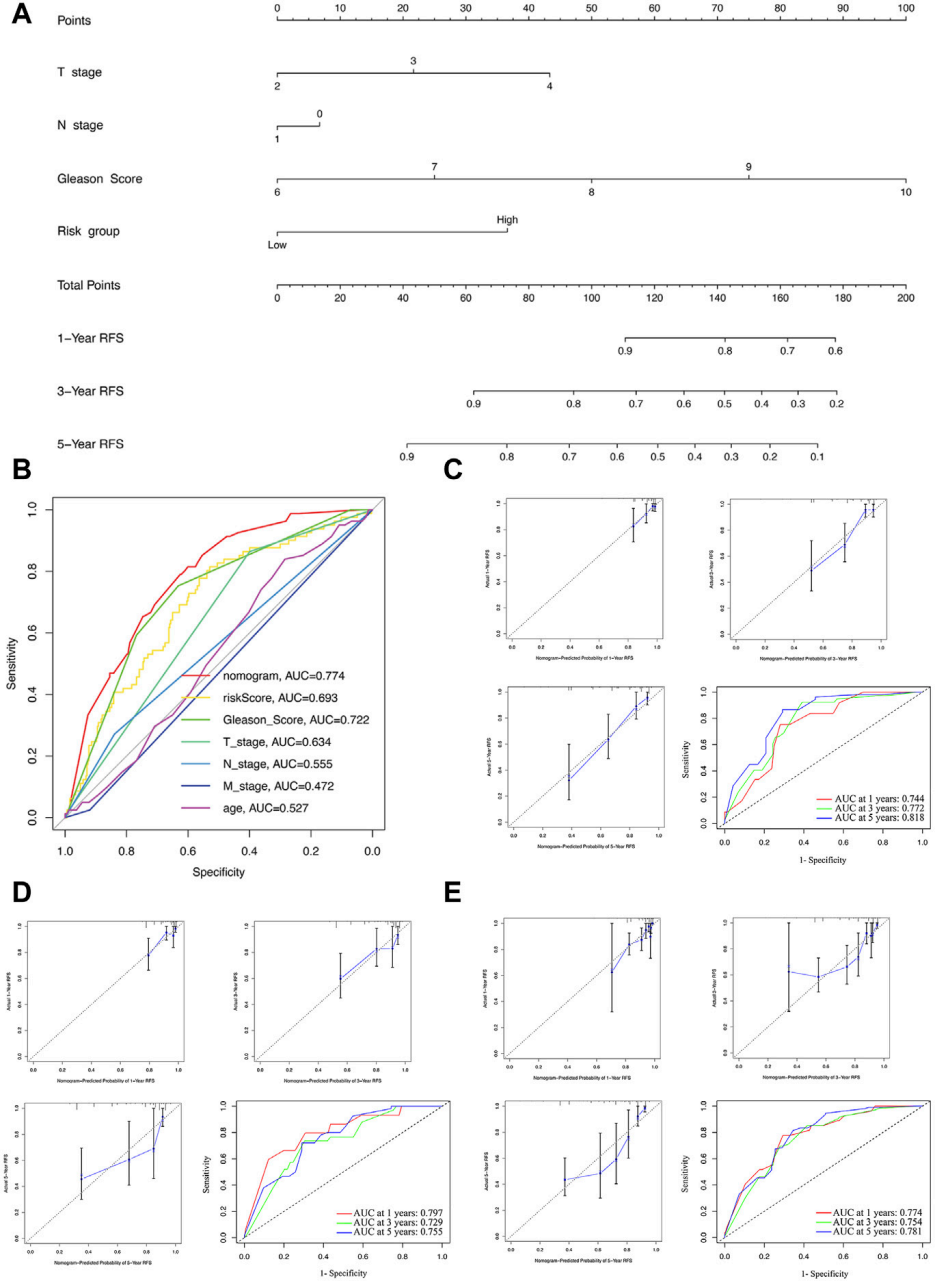
Construction and evaluation of the nomogram. **(A)** The nomogram combining the risk score and clinicopathological features. **(B)** ROC curve analysis of the nomogram, risk score and clinicopathological features. Evaluation of the nomogram model with calibration curves in 1, 3, and 5 years and ROC curves analysis in the training **(C)**, test **(D)**, and whole sets **(E)**.

In order to determine whether the risk score was an independent prognostic risk factor, we conducted Cox regression analysis. Figure 5G illustrated the results of univariate Cox regression analysis, which showed that the BCRFS was significantly correlated with T stage (HR = 2.557, *p* < 0.001), N stage (HR = 1.831, *p* = 0.016), Gleason score (HR = 2.208, *p* < 0.001) and risk score (HR = 3.580, *p* < 0.001). By performing multivariate Cox regression analysis, we found that only the risk score (HR = 2.340, *p* = 0.002) and Gleason score (HR = 1.789, *p* < 0.001) were independent prognostic risk factors for BCR of PCa patients (Figure 5H). Furthermore, the multivariate ROC analysis indicated that the AUCs of risk score was 0.693, higher than T stage and N stage; and just lower than Gleason score of 0.722 (Figure 6B). We also compared clinicopathologic variables, including age, TNM stage, and Gleason score between the high- and low-risk group. As shown in the heatmap (Figure 5I), it was found that T stage, N stage, and Gleason score were significantly different in the two groups.

### The nomogram construction and evaluation

For further predicting the BCRFS for PCa patients, an integrated nomogram was constructed, including the risk score and clinicopathological features (age, T stage, N stage, M stage, and Gleason score) to provide a clinically predictive tool for 1, 3, and 5-year BCR rate of all patients (Figure 6A). An evaluation of the effectiveness and accuracy of the nomogram was conducted in the training set, as well as a verification of its stability on the test and whole sets. An early assessment of BCR for PCa patients can be accomplished using this nomogram, which had the highest AUC value of 0.774 (Figure 6B). The calibration curve for 1-, three- and 5-year BCR displayed good uniformity between actual outcomes and the predicted BCRFS in the training set (Figure 6C). Based on time-dependent ROC analysis, our nomogram also displayed an excellent prediction performance for BCR in the training set, with the AUCs of 0.744, 0.772, and 0.818 for 1-, three- and 5-year BCR, respectively (Figure 6C).

To further validate the nomogram, the calibration curve displayed a good uniformity between actual outcomes and the predicted BCRFS time in the test and whole sets (Figures 6D,E). Meanwhile, the AUCs of the test set were 0.797, 0.729, and 0.755 (Figure 6D). For the whole set, the AUCs were 0.774, 0.754, and 0.781 (Figure 6E). In sum, our nomogram was excellent at predicting the BCRFS in PCa patients.

### Functional enrichment analysis

**FIGURE 7:**
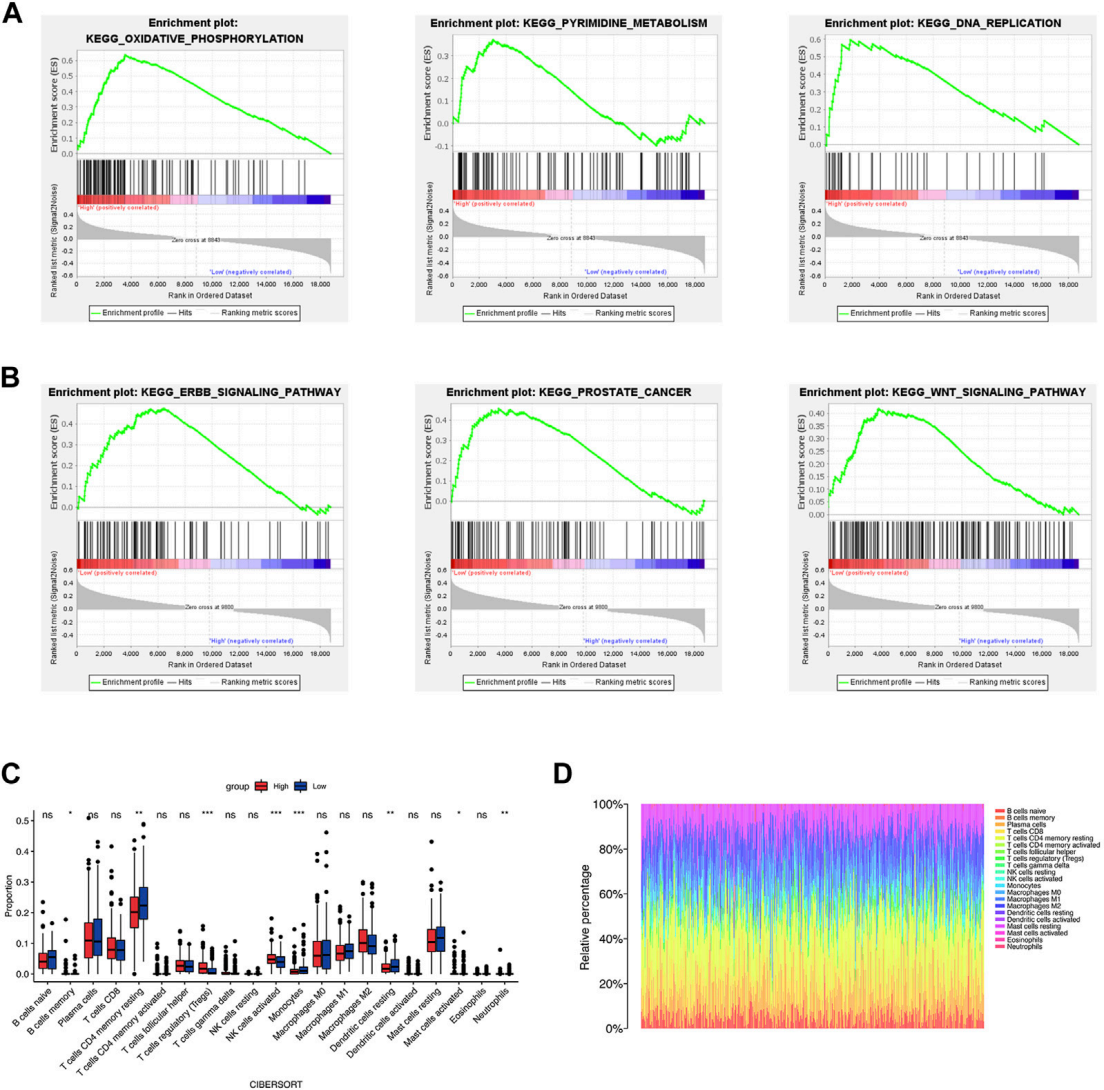
Functional enrichment analysis and immune cell infiltration. **(A, B)** The results of GSEA analysis of the high- and low-risk groups. **(C, D)** The immune cell infiltration by CIBERSORT algorithm. *p < 0.05; **p < 0.01; ***p < 0.001; ns, no significance.

We conducted GSEA to investigate the molecular mechanisms underlying significant differences between the two groups. The results (Supplementary Table S3) indicated that the high-risk group was significantly associated with oxidative phosphorylation, base excision repair, DNA replication, and pyrimidine metabolism (Figure 7A). The enriched pathways of the low-risk groups were ERBB signaling pathway, prostate cancer, and WNT signaling pathway (Figure 7B).

### Immune cell infiltration analysis

**FIGURE 8:**
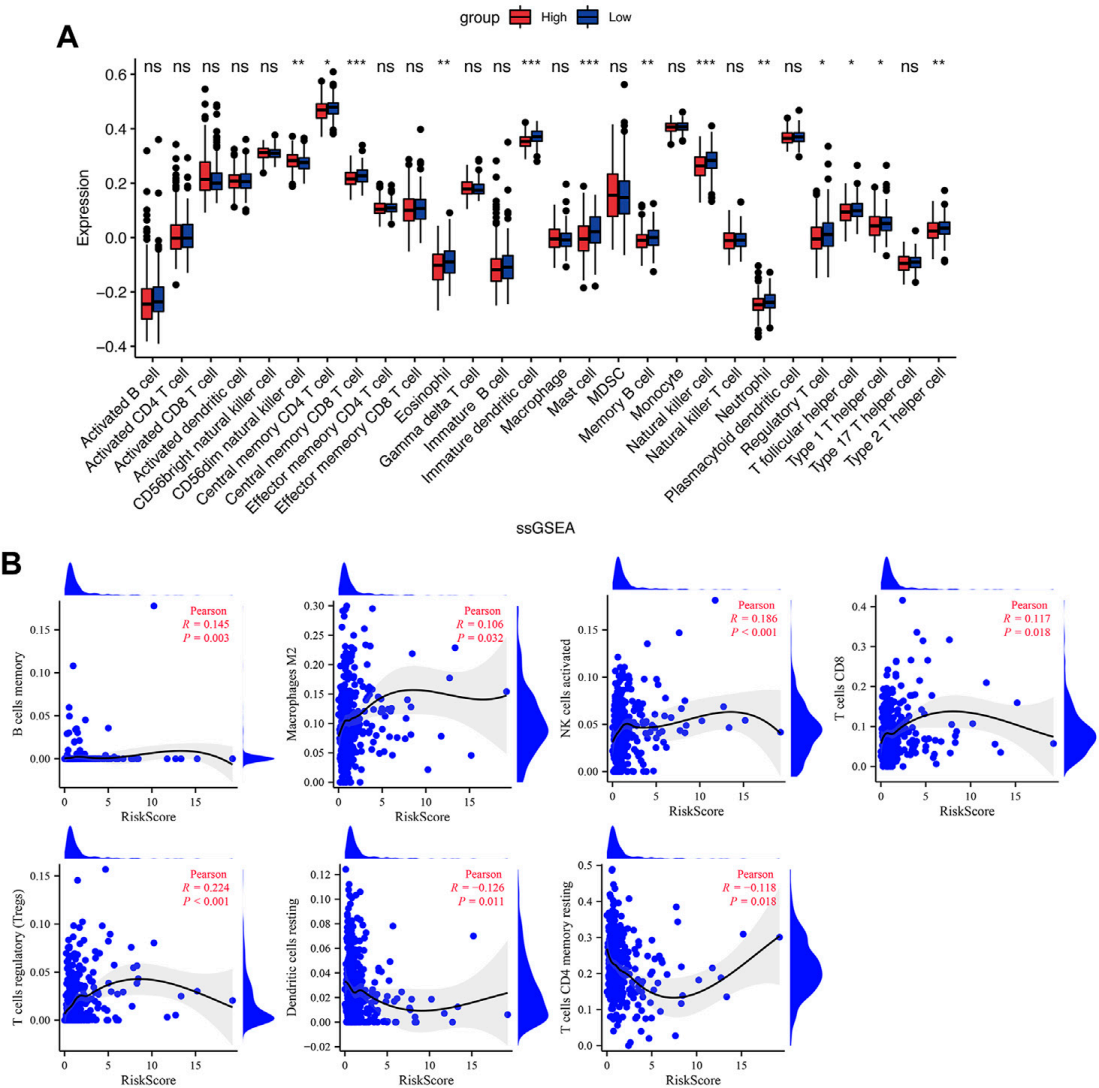
Immune cell infiltration in the low- and high-risk groups. **(A)** The infiltrating levels of immune cells in the low- and high-risk groups. **(B)** The correlation between risk score and immune cell subtype infiltration. *p < 0.05; **p < 0.01; ***p < 0.001; ns, no significance.

TME is critical for tumorigenesis and progression, as well as immune cell infiltration (Arneth, 2019). As a result, we compared the proportions of 21 immune cell types between the two groups to explore the correlations of the risk score and TME. According to CIBERSORT algorithm, B cells memory, T cells regulatory (Tregs), NK cells activated, and Mast cells activated had higher infiltrations in the high-risk group, while T cells CD4 memory resting, Monocytes, Dendritic cells resting and Neutrophils had higher infiltrations in the low-risk group (Figures 7C,D). The values of Pearson correlation were figured out between risk scores and the CIBERSORT calculated immune cells infiltration values. Further, we analyzed subgroups of the immune cell in the two groups using ssGSEA. It was found that the infiltration levels of central memory CD4^+^ T cell, immature dendritic cell, mast cell, memory B cell, NK cell, neutrophil, Treg, T follicular helper cell, type 2 T helper cell, Eosinophil, and central memory CD8^+^ T cell were lower in the high-risk group, while CD56dim NK cells and type 1 T helper cell were higher infiltrated (Figure 8A). As depicted in Figure 8B, the infiltration values of B cells, NK cells, CD8^+^ T cells, Macrophages M2, and Tregs were significantly positively correlated with the risk score, and the values of Dendritic cells and CD4^+^ T cells, and were significantly negatively correlated with the risk score.

### TMB analysis

**FIGURE 9:**
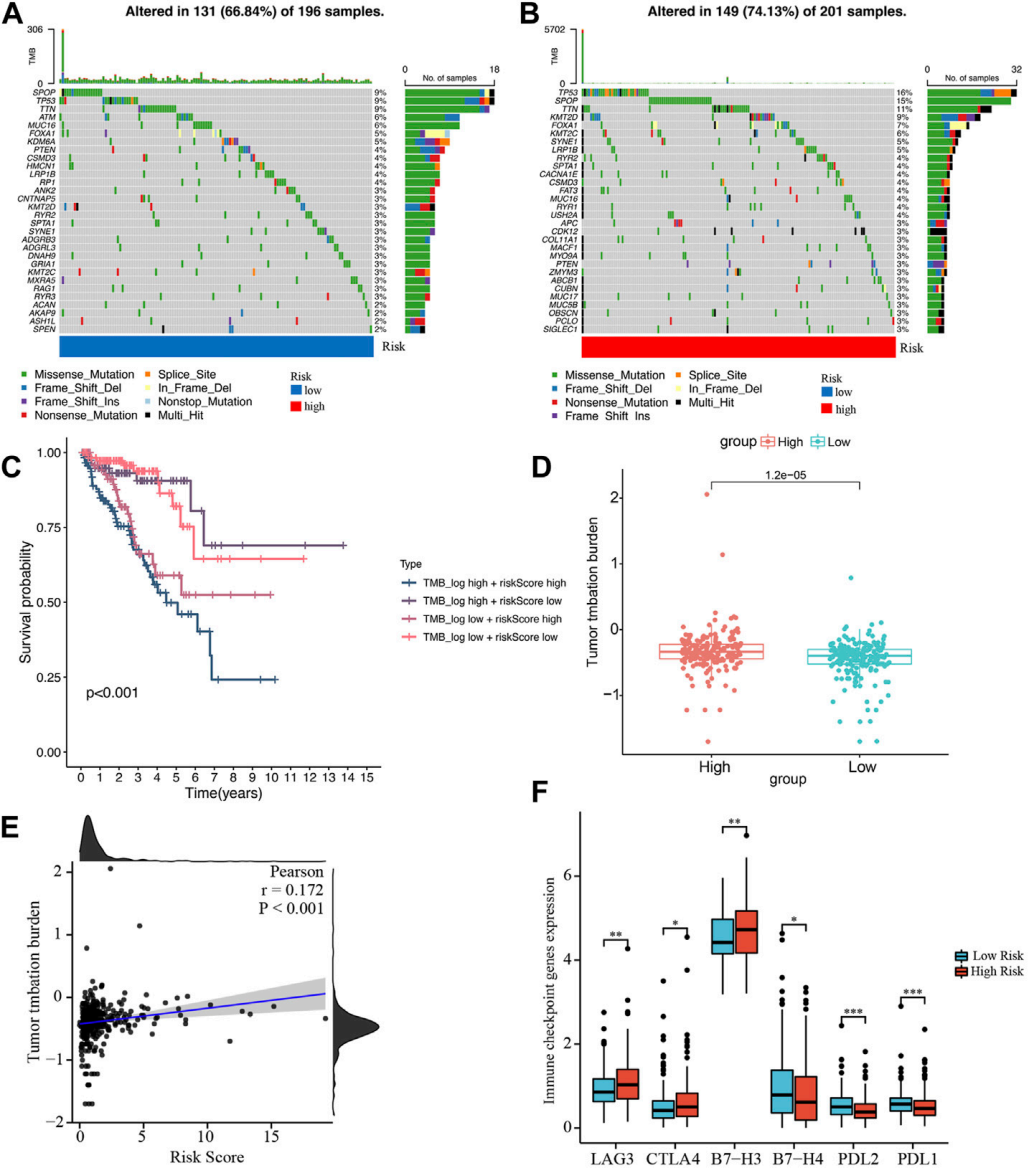
Analysis of tumor mutation burden and immune checkpoint genes. **(A, B)** Waterfall plots of mutated genes in the low- and high-risk groups, respectively. **(C)** Kaplan-Meier survival curve of BCRFS stratified by TMB and risk score. **(D)** The comparison of TMB between the low- and high-risk groups. **(E)** The correlation between TMB and risk score. **(F)** Differentially expressed immune checkpoint genes between the low- and high-risk groups. *p < 0.05; **p < 0.01; ***p < 0.001.

We obtained the somatic mutation data of each PCa patient from TCGA and analyzed the genetic alteration of the low- and high-risk groups. In Figures 9A,B, the top 10 most mutated genes differed between the two groups, among which SPOP and TP53 had the highest rate. In the high-risk group, patients with high TMB had the shortest BCRFS than other subgroups (Figure 9C). We found that the TMB in the high-risk group was remarkably higher than in the low-risk group, and the risk score was significantly positively correlated with TMB (Figures 9D,E).

### Prediction of the sensitivity to antitumor drug with the risk score

**FIGURE 10:**
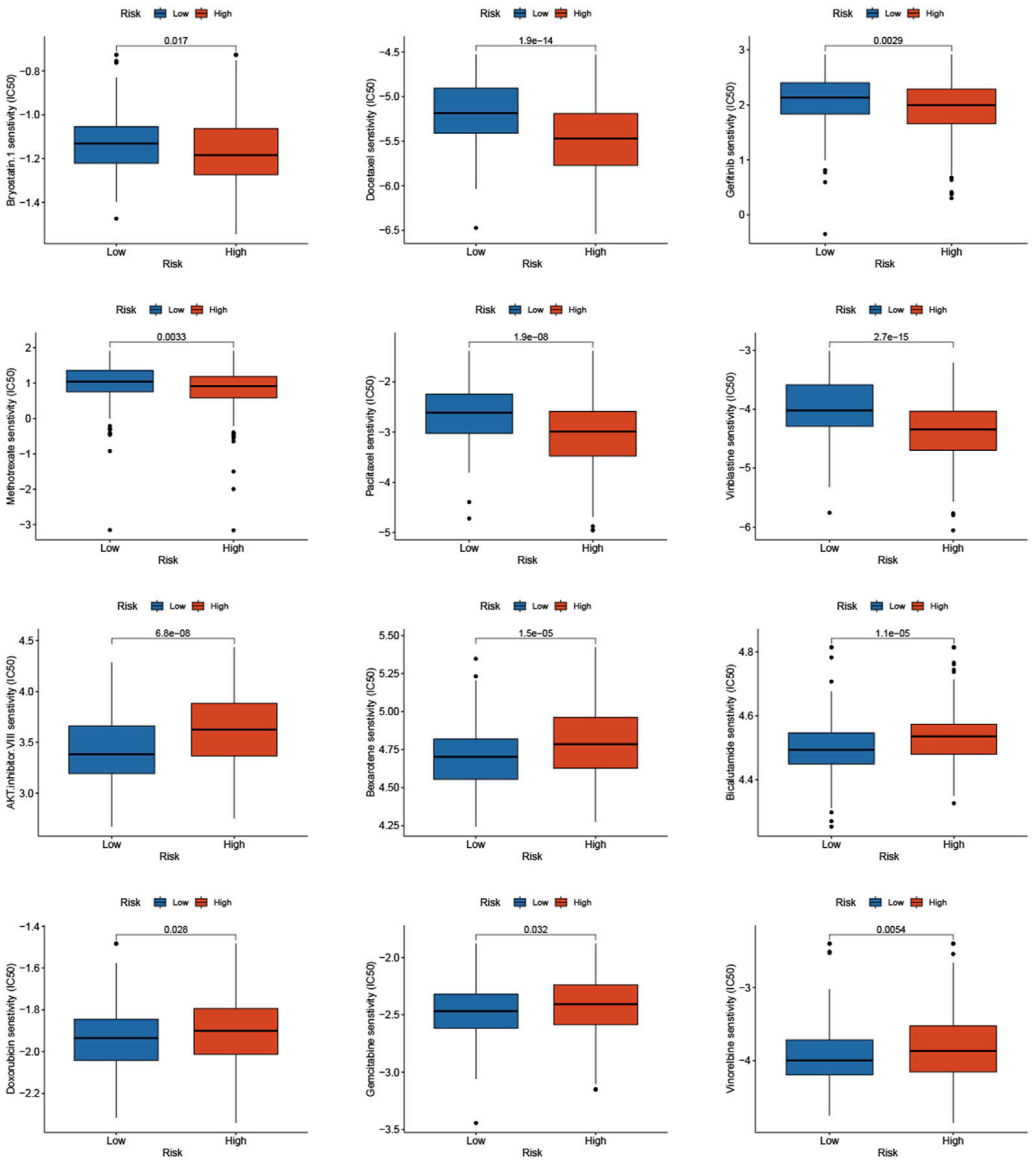
Prediction of the sensitivity to chemotherapeutic drug with the risk score by IC50 values.

Based on studies by Kgatle MM et al. (Kgatle et al., 2021), we collected 17 significant immune checkpoint genes and investigated their expression levels between the low- and high-risk groups. We found that the high-risk group patients had significantly higher expression of LAG3, CTLA4 and B7-H3, while patients in the low-risk group had higher expression of B7-H4, PDL-2 and PDL-1 (Figure 9F **)**. Then, we assessed the responses of the two groups to chemotherapeutic drugs by the half-maximal inhibitory concentration (IC50) values. The results showed that PCa patients in the high-risk group were more susceptible to bryostatin.1, docetaxel, gefitinib, methotrexate, paclitaxel, and vinblastine, whereas patients in the low-risk group were more susceptible to AKT inhibitor VIII, bexarotene, bicalutamide, doxorubicin, gemcitabine, and vinorelbine (Figure 10 **)**.

### Expression level of crlncRNAs in prostate cancer cell lines

**FIGURE 11:**
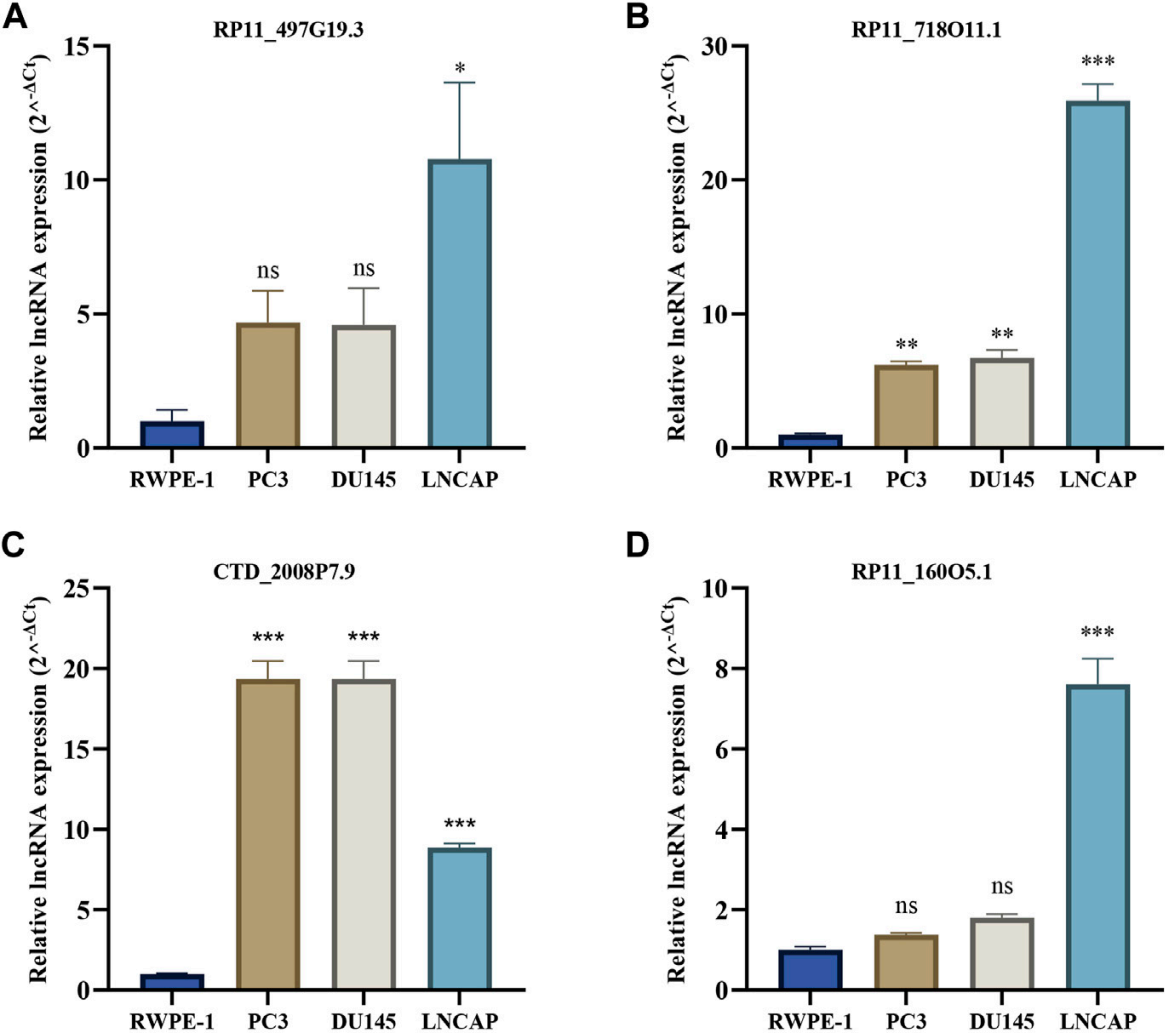
The qRT-PCR results of four crlncRNAs in three PCa cell lines. **(A)** qRT-PCR result of RP11-497G19.3. **(B)** qRT-PCR result of RP11O-718O11.1. **(C)** qRT-PCR result of CTD-2008P7.9. **(D)** qRT-PCR result of RP11O-160O5.1.

We further validated the expression levels of four crlncRNAs in PCa cells PC3, DU145, and LNCAP by qRT-PCR. As shown in Figure 11, we found that RP11_497G19.3, RP11_718O11.1, CTD_2008P7.9, and RP11_160O5.1 were significantly upregulated in PCa cell lines compared with those in the RWPE-1 cells. In our bioinformatics analyses, CTD_2008P7.9 was significantly downregulated in the PCa samples which is inconsistent with the results of qRT-PCR.

## Discussion

The early BCR of PCa patients after radical prostatectomy (RP) or radical radiotherapy (RRT) is associated with underlying clinical metastases and poor prognosis (Xu et al., 2020). Early detection and seasonable intervention for PCa patients with BCR can improve the clinical prognosis and outcomes. Therefore, accurate and effective prediction for early BCR and reasonable management strategies are essential. Recently, Tsvetkov et al. uncovered a new type of regulated cell death (RCD), cuproptosis, which arises from copper-induced lipoylated proteins aggregation and loss of iron-sulfur cluster protein in TCA cycle, resulting in proteotoxic stress regulated cell death (Tsvetkov et al., 2022). Mitobe Y et al. pointed out that lncRNAs play a special role in the development of PCa as well as progression to endocrine therapy resistance and would be promising therapeutic targets of advanced PCa (Mitobe et al., 2018). In a recent study, Liu et al. identified a ferroptosis-related lncRNAs signature consisting of five lncRNAs to predict the BCR of PCa patients (Liu C. et al., 2022). The purpose of this study was to establish a novel cuproptosis-related lncRNA (crlncRNA) signature for studying biochemical recurrence (BCR) and tumor immune landscape of prostate cancer (PCa).

It has been reported that ten cuproptosis-related genes are associated with the process of cuproptosis, including FDX1, LIPT1, DLD, LIAS, DLAT, PDHA1, PDHB, MTF1, GLS, and CDKN2A (Tsvetkov et al., 2022). Our present study found that only two of these genes, including CDKN2A and LIPT1, were differentially expressed between PCa samples and normal tissues. Lipoyltransferase 1, encoded by LIPT1, is a vital enzyme that transfers lipoate to the E2 subunits of the 2-ketoacid dehydrogenase complexes, and the deficiency of it had already been confirmed to suppress TCA cycle metabolism (Solmonson et al., 2022). Studies have shown that LIPT1 is closely linked to a favorable outcome in patients with urothelial cancer, hepatocellular carcinoma, and melanoma (Chen et al., 2021; Lv et al., 2022; Yan et al., 2022). In this study, we found that LIPT1 was upregulated in PCa, but not correlated with the BCR. CDKN2A, a tumor suppressor gene, encodes the p16^INK4a^ protein that negatively regulates the cell cycle, which is an important tumor suppressor protein. Loss of CDKN2A could promote tumorigenesis and metastasis and indicate a poor prognosis in many cancers, including PCa (Zhao et al., 2016). The study by Lu W et al. revealed that interaction between HNF1B with CDKN2A could play a crucial role in the development and progression of PCa (Lu et al., 2020). Furthermore, methylation of the CDKN2A promoter was associated with poor prognosis in PCa patients and was also closely related to the metabolism of copper ions in humans (Ameri et al., 2011; Silva et al., 2020). We found that the expression of CDKN2A was upregulated in PCa and was strongly correlated with poor prognosis. Thus, we can infer that CDKN2A, *via* the methylation of its promoter, is involved in the process of cuproptosis, which may play a vital role in PCa initiation and progression.

We obtained 455 samples with complete clinical data and RNA-seq from the TCGA database. In order to assess and quantify the cuproptosis-related lncRNAs of PCa patients, and accurately predict the BCRFS, we randomly divided all PCa patients into a training set and a test set in a1:1 ratio. By performing LASSO and multivariate Cox regression in the training set, we screened a total of six cuproptosis-related lncRNAs and constructed a crlncRNA signature prognostic model. We calculated risk scores of each patient according to the formula. All patients were divided into the low-risk and high-risk groups according to median value of risk scores. The survival curves revealed that patients in the high-risk group tended to have significantly shorter BCRFS than in the low-risk group.

We utilized the ROC curves analysis to identify that the prognostic model had good accuracy in predicting the BCRFS in the training set, which was validated in the test set and whole set. Our results showed that the risk score was an independent prognostic factor for the BCR of PCa patients. We then constructed an integrated nomogram, including risk score, age, T stage, N stage, M stage, and Gleason score. ROC curves indicated that the nomogram showed a good performance in predicting the BCRFS. The calibration curve displayed good uniformity between actual outcomes and the predicted BCRFS time.

Our cuproptosis-related lncRNA signature contained six lncRNAs: RP11-497G19.3, RP11-575F12.3, RP11O-718O11.1, MIR100HG, CTD-2008P7.9, and RP11O-160O5.1. By performing multivariate Cox regression, we found that RP11O-160O5.1 was an independent prognostic risk factor for BCR of PCa patients and we inferred that it might be a prognostic indicator for PCa patients. LncRNA MIR100HG was dysregulated in many cancers and played distinctively complex and contradictory roles, which involved tumorigenesis, proliferation, and invasion or inhibition of these biological behaviors of tumors (Wu et al., 2022). Previous studies had demonstrated that lncRNA MIR100HG was identified as an oncogene in various cancers, including breast cancer (Chen et al., 2020), hepatocellular carcinoma (HCC) (Li et al., 2021), gastric cancer (Li J. et al., 2019; Li et al., 2020), and colorectal cancer (Li W. et al., 2019). However, studies in MIR100HG also proved that it could exert tumor suppressive effects in papillary-thyroid carcinoma (Yang et al., 2021; Park and Lee, 2022) and cervical cancer (Lang et al., 2022; Lin et al., 2022). Furthermore, there are opposing results about the role of lncRNA MIR100HG in bladder cancer (BC). Ying W et al. found that MIR100HG is lowly expressed in BC, and the overexpression of MIR100HG inhibits the proliferation and invasion of BC cells (Ying et al., 2021). Conversely, it had been reported from Zhang S’s study that the overexpression of MIR100HG effectively promoted the proliferation, migration, and invasion of BC cells, and it was proved to be an independent prognostic factor for BC (Zhang et al., 2021). Our results suggested that MIR100HG was lowly expressed in PCa and indicated that this lncRNA might play a tumor-suppressive role in PCa. For the other five lncRNAs, no relevant studies in tumors were reported, which may provide us with new research perspectives to further improve the predictive ability of BCR for PCa patients and explore underlying mechanisms between cuproptosis and PCa.

It has been reported that innate immune cells and adaptive immune cells played an important role in the proliferation and invasion of tumors in the tumor microenvironment (TME) (Hinshaw and Shevde, 2019). Therefore, we utilized ssGSEA to quantify differences in the immune cell infiltration between the high- and low-risk groups, then further explored the association of the risk score and immune status. The results revealed that patients in the high-risk group presented reduced infiltration levels of central memory CD8^+^ T cells (TCM) and NK cells, which was highly consistent with previous studies that both TCM and NK cells play a major role in effective anti-tumor responses (Fridman et al., 2017; Bohner et al., 2019). We also found that infiltration levels of mast cells were evaluated in the low-risk group. Johansson A et al. demonstrated that highly infiltrated mast cells in PCa tissue could inhibit tumor growth and development and indicate a better prognosis (Johansson et al., 2010). These results suggested that this crlncRNA signature may play a vital role in tumor proliferation and invasion *via* regulating the TME, especially TCM, NK cells, and mast cells. Therefore, targeting cuproptosis is expected to be a novel therapeutic strategy to suppress PCa progression or enhance the effect of castration therapy.

We also found a significant difference in genetic mutation between the two groups, and the risk score was positively correlated with TMB. PCa patients in the high-risk group with high TMB had shorter BCRFS, indicating that high TMB was correlated with poor prognosis. TP53 and SPOP had the highest mutated rate in the low- and high-risk groups, respectively. The mutation of SPOP could cause genomic instability as an early event that drives tumorigenesis in PCa and increase sensitivity to DNA-damaging therapeutics (Boysen et al., 2015). The TP53 mutation was the most mutated gene in PCa patients and had been demonstrated to involve progression and metastasis in PCa (Xu X. et al., 2022; Maxwell et al., 2022). Due to increasing attention to immune checkpoint inhibitors for PCa patients, we analyzed differentially expressed immune checkpoint genes between the two groups and found that patients in the high-risk group tended to express higher levels of LAG3, CTLA4, B7-H3, and B7-H4. It had been reported that high levels of immune checkpoints and TMB predicted improved effectiveness of immunotherapy (Wu et al., 2019; Fahmy et al., 2021). We demonstrated that the high-risk group patients had higher TMB and the risk score is significantly positively correlated with TMB, which indicated that patients with high risk score may benefit more from immunotherapy. Then, we assessed the sensitivity of chemotherapeutic drugs in the two groups. The results revealed that PCa patients in the high-risk group were more susceptible to bryostatin.1, docetaxel, gefitinib, methotrexate, paclitaxel, and vinblastine, whereas patients in the low-risk group benefited more from AKT inhibitor VIII, bexarotene, bicalutamide, doxorubicin, gemcitabine, and vinorelbine. Therefore, the risk model could guide clinicians to provide PCa patients with individual-based treatment regime.

However, wo do have a few limitations with this study. Firstly, the crlncRNA prognostic model was constructed and validated only using the TCGA database. Therefore, more external databases are needed to validate its prognostic significance. Secondly, our analysis refers to a retrospective analysis of public data, and selection bias is inevitable in it that may affect the precision and accuracy of our results. Finally, further experimental proof (*in vivo* or *in vitro*) is essential to uncovering molecular mechanisms of crlncRNAs regulating the progression and invasion of PCa.

## Conclusion

This study was to identify a novel cuproptosis-related lncRNA signature to precisely predict BCR of PCa patients based on risk scores. We demonstrated that the risk score was an independent prognostic factor and identified significant differences in immune cell infiltration and TMB between the low- and high-risk groups. Our results can assist researchers further understand the role of cuproptosis in PCa and provide new insights into developing individual-based treatment strategies.

## Data Availability

Publicly available datasets were analyzed in this study. This data can be found here: https://portal.gdc.cancer.gov/repository.

## Supplementary material

Supplementary Figure S1 The forest plot of 14 prognostic lncRNAs.

Supplementary Figure S2
**(A-C)** Kaplan-Meier survival analysis of BCR in the low- and high-risk groups for patients with T2, GS>65, and N1.

Supplementary Table S1 Primers for qRT-PCR.

Supplementary Table S2 101 differentially expressed cuproptosis-related lncRNAs.

Supplementary Table S3 GSEA analysis of enriched pathways in the high- and low-risk groups.

## Abbreviation

lncRNA, long non-coding RNA; crlncRNA, cuproptosis-related lncRNA; DE-crlncRNAs, differentially expressed cuproptosis-related lncRNA; PCa, prostate cancer; BCR, biochemical recurrence; BCRFS, biochemical recurrence free-survival; TMB, tumor mutation burden; TME, tumor microenvironment; DEGs, differentially expressed genes; RCD, regulated cell death.
