# Supplementary figures and images for: A cuproptosis-related LncRNA signature: Integrated analysis associated with biochemical recurrence and immune landscape in prostate cancer
Source: Front Genet. 2023 Feb 24;14:1096783. doi: 10.3389/fgene.2023.1096783 (PMC9999016; doi:10.3389/fgene.2023.1096783)

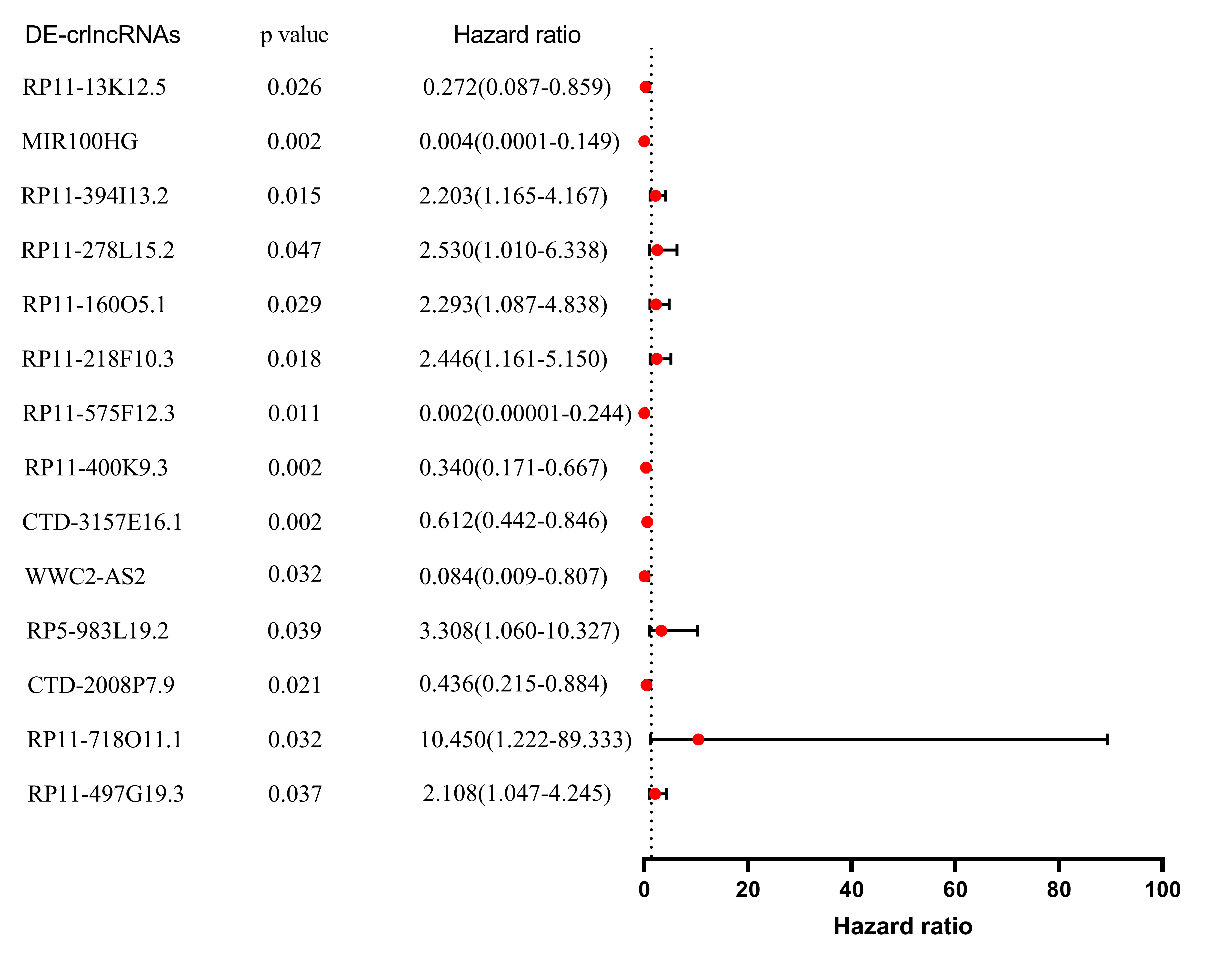

Supplement: Supplementary file 4 [file Image1.TIF]
